# Multi-Scale Assessment of Photo-Oxidative Versus Thermal Aging in Extracted Layered Cores and Un-Extracted Bitumen Films

**DOI:** 10.3390/ma19214498

**Published:** 2026-10-26

**Authors:** Ahmed Abouelsaad, Greg White

**Affiliations:** 1 Faculty of Engineering, Mansoura University, Mansoura 35516, Egypt; a_m_naguib@mans.edu.eg; 2 Faculty of Engineering, Mansoura National University, Gamasa 35712, Egypt; 3 School of Science, Technology, and Engineering, University of the Sunshine Coast, Sippy Downs, QLD 4556, Australia

**Keywords:** asphalt, aging, accelerated, laboratory, thermal oxidation, photo-oxidation

## Abstract

Asphalt pavements experience complex, depth-dependent aging driven by combined thermal-oxidative and UV photo-oxidative mechanisms. Current laboratory aging protocols often evaluate either only binder films or bulk mixtures exposed to thermal or photo oxidation; that is why this study aimed to contrast the differences in the chemical and rheological behaviors of both bitumen extractions and thin bitumen films exposed to heat only and heat combined with UV irradiation. Three dense-graded asphalt mixture samples were aged in an oven at 70 °C. For comparison, three samples with the same properties were aged in an ultraviolet (UV) weathering chamber at the same temperature but with UV irradiation at an intensity of 50 W/m^2^ for 98 days. Afterwards, samples were cut into three disks, and binder extractions from the top and middle disks were tested using a dynamic shear rheometer (DSR), saturates–aromatics–resins–asphaltenes (SARA) analysis, and Fourier-transform infrared spectrometry (FTIR) analysis. Samples of the same bituminous binder were also applied to steel plates at a thickness of 1 mm before being aged and tested under the same conditions. The results showed that UV irradiation plays a significant role in the aging process of bituminous binders in both asphalt mixtures and bitumen samples. In terms of DSR and SARA analysis results, extractions from the top disks of the UV- and oven-aged mixtures showed far more severe aging compared to the bituminous binder films, while the effect of pavement depth was found to have a more significant effect in samples that had been aged in the UV chamber. FTIR results followed the same trend, but the bituminous binder results were comparable to the results of the extractions from the aged mixture samples. It was concluded that binder aging research is better performed using binder specimens extracted from aged asphalt mixture samples, rather than thin films of binder, and that UV irradiation is essential for more realistic accelerated aging protocols.

## 1. Introduction

Flexible pavements are engineered multi-layered viscoelastic structures designed with high load-bearing capacity [1] to keep strain levels at subgrade and lower asphalt layers controlled below the critical strain levels defined by modern mechanistic–empirical principles, including the Perpetual Pavement framework, to prevent deep structural rutting and bottom-up fatigue cracking. Although the theoretical design life of asphalt pavements is generally governed by subgrade rutting and asphalt fatigue cracking [2], in practice, distresses such as fretting and raveling, caused by the loss of mastic bridges between coarse aggregate particles due to binder aging [3,4], often trigger resurfacing. Hence, understanding the asphalt binder aging phenomenon is critical to pavement researchers. It follows that developing a realistic protocol that accelerates aging in the laboratory and realistically simulates field aging conditions has gained the interest of many researchers lately. Consequently, understanding the effects of different environmental factors, including heat and ultraviolet (UV) irradiation, on binder and asphalt concrete mixtures is considered a mandatory step toward developing the proposed protocol.

Exposure to the environmental conditions causes binder aging, which is defined as changes in the chemical and rheological properties of the binder with time [5]. Binder aging can be caused by oxidation due to a reaction between heat and air, which is known as thermal-oxidative aging [6,7,8], or photo-oxidative aging, which is caused by ultraviolet (UV) irradiation and oxygen [9]. Both heat and UV light can degrade the bitumen properties, but their effects on bitumen aging are different [10]. For example, Wang et al. [11] found that a more complicated aging behavior is noticed when heat and UV irradiation are combined. Thermal-oxidative aging has specific laboratory simulation standards, including the Rolling Thin-Film Oven (RTFO) [12] and Pressure Aging Vessel (PAV) [13]. The RTFO is intended to simulate aging during the production and placement of asphalt mixtures, while the PAV is intended to simulate long-term field aging (7–10 years). In contrast and despite its recognized importance, most standard specifications for routine binder grading such as Superpave do not currently incorporate UV conditioning [14] and rely only on elevated temperature and pressure to accelerate aging.

Many studies have been carried out to investigate the different effects of UV irradiation on the rheological and chemical properties of bitumen. For example, Menapace et al. [15] found that combining UV irradiation and heat resulted in more significant chemical changes in the binder compared to RTFO and PAV standard protocols. Similarly, Kuang et al. [16] concluded that the combination of thermal and UV aging had a different effect on binder properties. Other researchers have also confirmed the significant effect of UV irradiation on the rheological properties of different binders and its role as a photochemical initiator in the aging process [17,18,19,20].

Regarding asphalt binder aging, some researchers believe that neither the RTFO, nor the PAV, is sufficient to simulate field aging of binders. First, neglecting UV irradiation as a contributing factor to the aging phenomenon can be a limitation of current protocols [15,21,22,23]. Second, applying the same protocols despite variability in climatic conditions between different regions is unrepresentative and inappropriate [5]. Third, binder aging protocols cannot account for any interaction between the binder phase and the aggregate matrix, which occurs in asphalt concrete mixtures. The presence of aggregates affects binder film thickness, oxygen diffusion pathways, and the chemical aging mechanisms of the binder, resulting in aging behavior that may differ from that observed in isolated binder samples [24,25,26].

In terms of the aging of compacted asphalt concrete specimens, AASHTO R30 [27] is the most common standard used, in which compacted mixture samples are conditioned at 85 °C for five days, intended to simulate five to ten years of field aging, evaluating pavement field sections across various climatic regions in the United States [28]. However, using a single time-temperature combination is not universally appropriate for all climatic conditions, and many studies have found that the extracted binder from the aged asphalt concrete was less aged than that recovered from field coring of aged existing pavement surfaces. In the NCHRP 09-54 report [28], it was found that conditioning loose mixture samples at 85 °C for 41 days is required to achieve the same degree of aging as eight years in the field, while 21 days is required to achieve the same eight years of field aging at 95 °C. In a similar study, Jing et al. [29] concluded that the AASHTO long-term aging protocol is far less severe than the actual field conditions of even a three-year-old pavement surface in mild climatic conditions.

Another limitation of conditioning compacted mixture samples at high temperature is the physical distortions of the specimens that can occur due to prolonged exposure to heat, which may be reflected in changes in air voids and sample geometries [30]. This is less of an issue when conditioning loose mixtures, rather than compacted samples, or conditioning thin disks cut from compacted mixture samples.

Regardless of the specimen used, aging affects the chemical and rheological properties of the asphalt binder. Hence, it is common to characterize the aging degree based on changes in rheological and chemical test parameters, including the results from a dynamic shear rheometer (DSR), Fourier-transform infrared spectroscopy (FTIR), and, more recently, the relative portions of saturates, aromatics, resins, and asphaltenes, known as SARA analysis. Many researchers have suggested using the DSR calculated complex shear modulus of binder samples, before and after aging, as an indicator of the degree of aging [31,32,33]. In terms of chemical properties, FTIR spectroscopy has been widely used as an indicator of the aging behavior of binders [34,35], and SARA analysis is also considered a reliable indicator of binder aging [36].

Some researchers have recently developed different aging protocols. However, there is no universal agreement on whether it is more appropriate to age the binder alone or to subject the entire asphalt concrete mixture to aging. Thus, there is an ongoing debate in the literature, with some researchers standing for binder aging protocols, while others believe that a mixture aging protocol is more appropriate. The researchers who advocate binder aging protocols state that controlled and reproducible laboratory conditions, with reliable and comparable standards such as RTFO and PAV, lead to rapid assessment of binder rheological and chemical changes. However, the binder-level protocols do not account for the interactions between the binder and aggregate, including diffusion effects and air void structures, which play a major role in aging [37]. In addition, mixture-level aging protocols are supported by performance-based testing approaches [38].

A key gap in current research is the lack of direct comparisons between binder aging and mixture aging of otherwise identical liquid asphalt materials. As a result, the predictive reliability of binder-only tests versus mixture-based protocols remains unclear [39,40,41]. Filling this gap will provide the foundation for more accurate, practical, and standardized methods to predict pavement durability. To address this gap, the present study systematically evaluated binder and mixture aging under identical material conditions, providing insight into their relative reliability for predicting pavement performance. In addition, towards a more realistic aging procedure, this study determined the different effects of heat only (thermal-oxidation), compared to the effect of combining heat and UV radiation (photo-oxidation), on the chemical and rheological properties of a thin film of binder, compared to extracted binders from a dense-graded airport asphalt concrete sample, compacted in the laboratory using a Superpave gyratory compactor, prior to simulated mixture aging. The DSR-based complex shear modulus, FTIR parameters, and SARA analysis were all considered as potential indicators of binder aging. The results were analyzed to consider three questions. First, what is the effect of thermal-oxidative aging (heat) of asphalt mixture binders, with and without photo-oxidative aging (UV irradiation)? Second, what is the importance of depth within the asphalt mixture sample on the aging of the binder? Finally, how does the aging of a thin film of bituminous binder compare to that of binder in a compacted asphalt mixture sample.

To conclude, in order to bridge the research gaps discussed and provide responses to the previously presented research questions, this study focused on adapting a dual approach, including the aging of thin films and compacted asphalt mixtures, to isolate the true chemical severity of surface photo-oxidation from aggregate-shielded depth gradients. By systematically contrasting depth-wise photo-oxidative penetration in compacted mixtures against unshielded binder film kinetics, this study advances the state of the art by clarifying the distinct roles of aggregate energy diffusion and surface photo-oxidation in laboratory accelerated aging.

## 2. Materials and Methods

### 2.1. Materials

A bulk sample of loose dense-graded asphalt (DGA) was sampled during the resurfacing of Emerald Airport, which is located in Queensland, Australia [42]. A bulk sample of the same asphalt binder was also retained from the same work. The asphalt sample was used to prepare six DGA specimens that were compacted using a Superpave gyratory compactor (Servopac, IPC Global, Boronia, VIC, Australia) to reflect the as-constructed density of the new runway surface. Three specimens were aged in a conventional oven at 70 °C for 98 days, while the other three specimens were aged at the same temperature, with the addition of UV irradiation, to understand the effect of UV light as a photochemical initiator on the bitumen degradation process. According to a previous study, the 98-day UV chamber protocol achieves an aging effect comparable to what would typically take 20 to 24 years under field conditions [43].

The asphalt mixture had volumetric compositions meeting the requirements of the Australian airport asphalt specification, as detailed in Table 1, and A10E polymer-modified binder was used, with its properties summarized in Table 2. A10E is highly modified with SBS polymer and is commonly used for airport pavement surfacing and other heavy-duty asphalt production in Australia [34].

**Figure 1:**
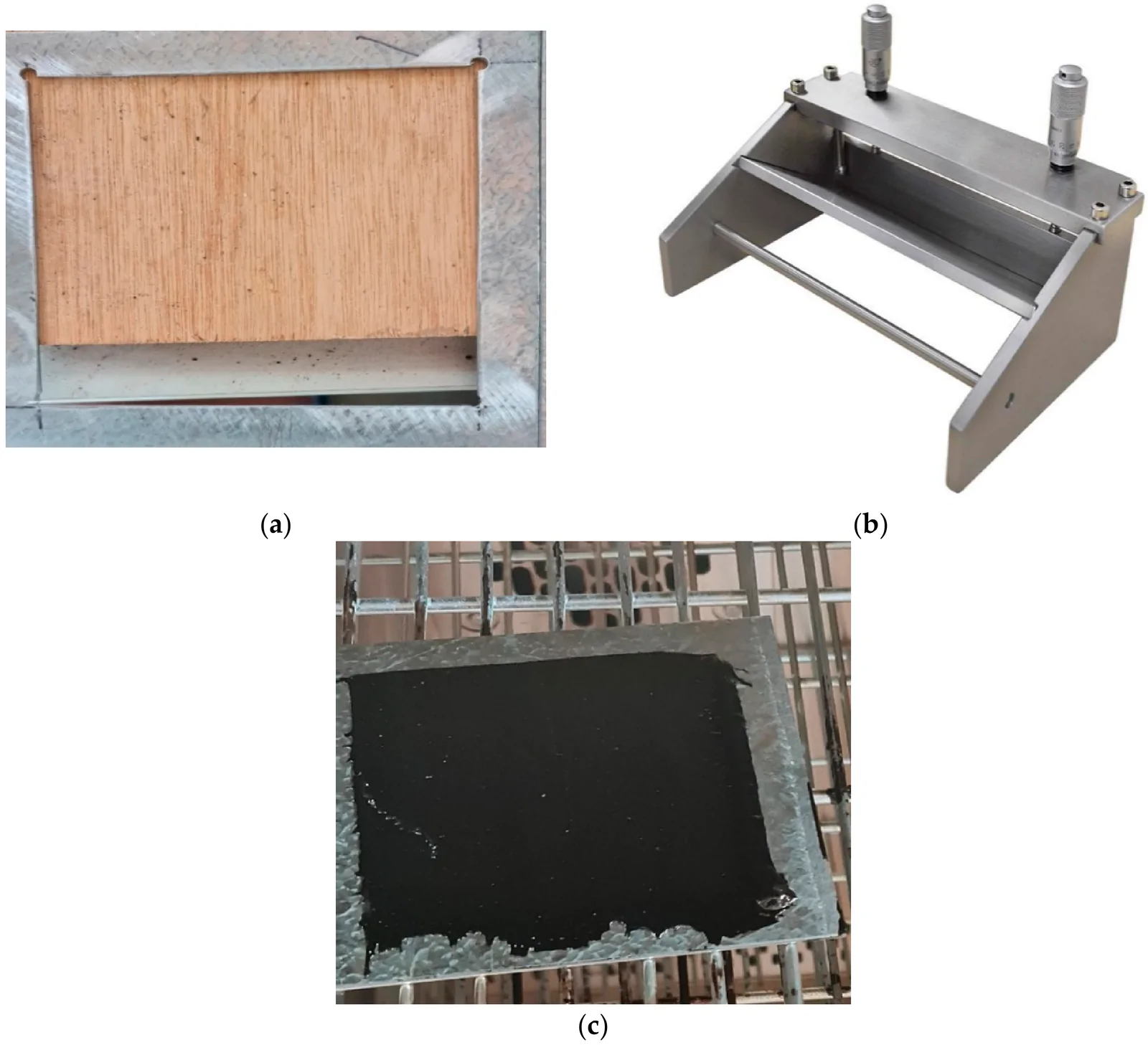
Bitumen thin film: (**a**) steel plate cutout, (**b**) applicator, and (**c**) example of specimen [45].

At the same time, 1 mm thick samples of the same binder were applied to steel plates using a hollow frame plate, and the thickness was verified using a 180 mm wet film applicator with a slurry guiding plate (Model EQ-Se-KTQ-180, MTI Corporation, Richmond, CA, USA), as shown in Figure 1. The thin-film binder samples were aged in the oven and the UV chamber for eight days; before the same testing, unaged samples were also tested as controls. The selection of 8 days was based on comparison with Zhang et al. [44], who subjected ~3.2 mm thick binder film samples to 12 days of UV aging at 8 W/m^2^ and 60 °C, whereas the thinner 1 mm films in this study, exposed to 50 W/m^2^ UV light at 70 °C, were expected to age more rapidly, making 8 days approximately equivalent to the 98-day mixture protocol, again representing 20–24 years of field aging [43].

### 2.2. Experimental Work

#### 2.2.1. Aging Procedures

As stated above, the primary purpose of this research was to compare the aging behavior of binder samples to that of mixture samples, under both thermal-oxidative aging and photo-oxidative aging, using the rheological and chemical properties of the binder as the indicator of aging. It should be emphasized that the 8-day aging of 1 mm thin binder films and the 98-day aging of 150 mm mixture cores are not assumed to be strictly equivalent in total oxidative dosage but to contrast the changes in the chemical and rheological behaviors of a thin film of bitumen and bitumen shielded by an aggregate structure in the compacted mixtures.

**Figure 2:**
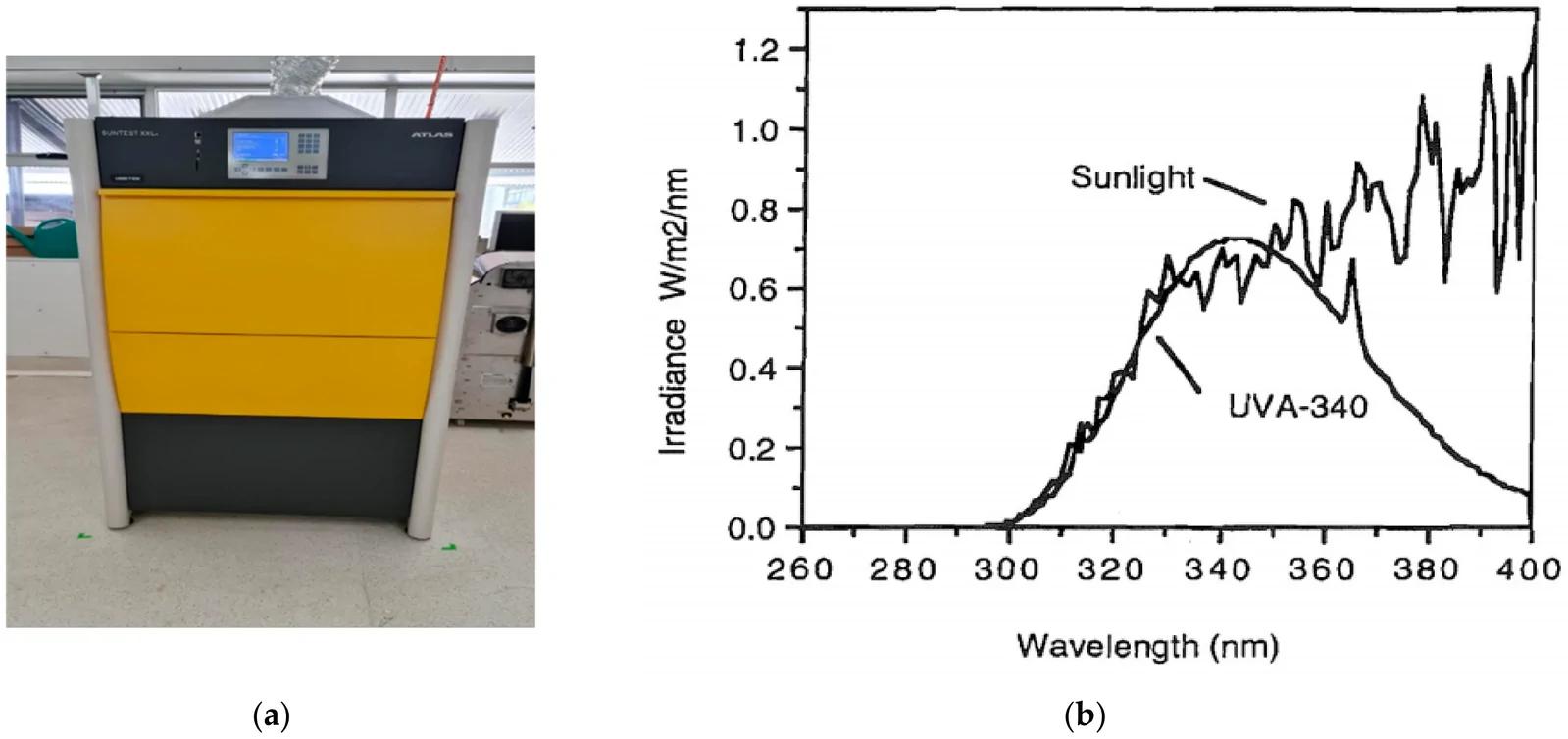
(**a**) Atlas Suntest XXL and (**b**) comparison of CIE85 sunlight and UVA lamp radiation [45].

A commercial Suntest XXL UV weathering chamber (shown in Figure 2) (Atlas Material Testing Solutions, Mount Prospect, IL, USA) was used to simulate photo-oxidative aging. The Suntest uses xenon lamps to simulate sunlight according to the CIE85 reference for sunlight, with an irradiance range of 30–65 W/m^2^ and control of irradiance either from 300 nm to 400 nm or 340 nm as a single representative wavelength. This more realistic simulation of sunlight over a wide range of wavelengths is a unique feature of the Suntest, compared to traditional fluorescent lamps. It can simulate the short-wave UV (<365 nm) portion of the spectrum [33], as shown in Figure 2. The Suntest weather settings are shown in Table 3.

#### 2.2.2. Cutting Samples and Extractions

**Figure 3:**
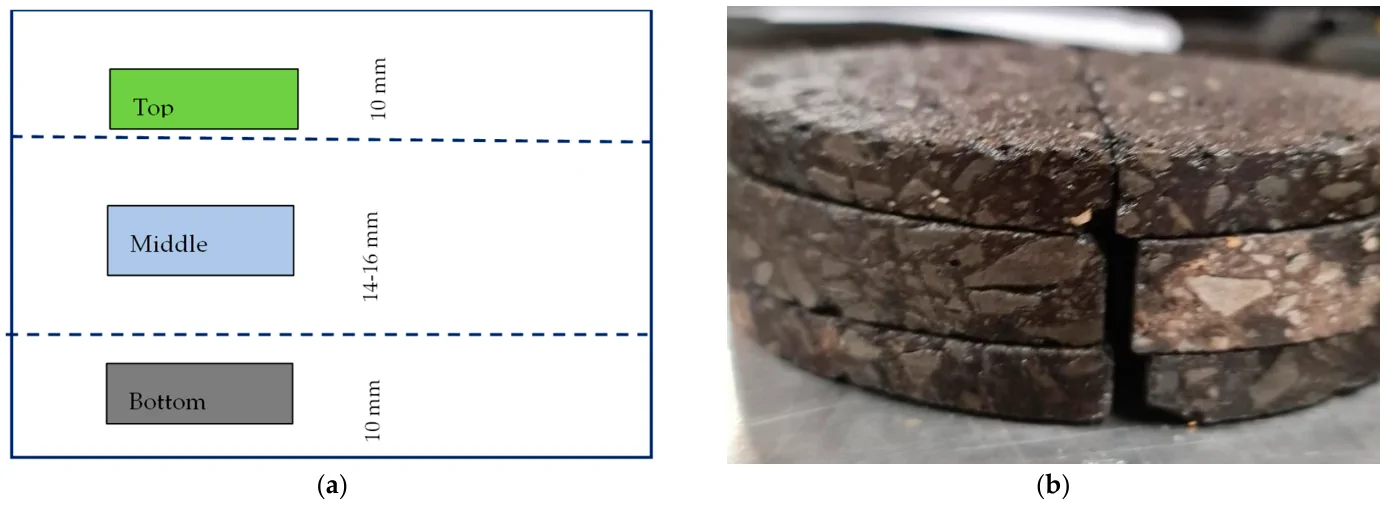
Core cutting plan: (**a**) schematic and (**b**) example [45].

After aging, each asphalt mixture specimen was cut into three disks, referred to as the top, middle, and bottom. The top and bottom disks were cut to a nominal thickness of 10 mm, with the middle disk having the remaining thickness after losses, approximately 15 mm, depending on the initial thickness of the core and the losses while cutting. A schematic and an example of the cutting stages are shown in Figure 3.

The asphalt binder was extracted from each disc. The bottom disks were intentionally discarded because they sit directly on a thermal conductive base, resulting in unrealistic thermal aging behavior compared to an asphalt surface in the field. Including binder extracted from this boundary disk would introduce confounding thermal effects that do not reflect true field environmental exposure [46]. The three specimens from each aging method were tested separately to confirm the repeatability of the results, with a total number of 12 extracted binder samples. Binder extraction involved heating the asphalt disks to 160 °C to deform the specimens into a loose mixture. After cooling, the loose mix was placed in toluene for two hours to dissolve the binder, which was then centrifuged for 15 min to remove any fine aggregate before the binder was recovered from the solvent using a rotary evaporator at the Fulton Hogan Testing Laboratory (Sydney, NSW, Australia).

For the asphalt binder specimens, no extraction of cutting was required. The aged thin film of binder near the middle of each specimen was isolated from the steel plate to avoid edge effects before testing.

#### 2.2.3. Bituminous Binder Testing

To evaluate the effect of aging on the extracted and thin-film binder specimens, three test methods were used:

- DSR frequency and temperature sweeps;
- SARA fractions;
- FTIR spectroscopy.

The DSR plays a crucial role in assessing the changes in rheological properties of asphalt binder due to aging, as it characterizes changes in stiffness and viscoelastic behavior. The increased modulus and reduced phase angle observed in aged binders demonstrate their loss of flexibility. Numerous researchers have widely employed the DSR to evaluate the rheological changes that asphalt binders undergo as a result of thermal and photodegradation. DSR frequency sweeps were performed from 0.1 to 10 Hz using a Kinexus DSR (NETZSCH-Gerätebau GmbH, Selb, Germany). The testing temperature ranged from 30 °C to 70 °C.

On the other hand, the importance of SARA analysis in aging studies lies in its ability to track binder composition shifts. Oxidation-induced changes, such as reduced aromatics and increased asphaltenes, are directly linked to hardening. As such, SARA analysis provides a chemical-level understanding of aging progression. Numerous studies have examined the changes in chemical fractions. Mirwald et al. [47] studied the effect of aging on SARA fractions and concluded that regardless of the aging technique used, similar changes in the four SARA fractions have been recorded. An increase in asphaltenes accompanies a significant loss in aromatics and resin content. The SARA analysis was performed using an Iatroscan thin-layer chromatography and flame ionization detection (TLC-FID) device (Iatroscan MK-6s, Iatron Laboratories, Inc., Tokyo, Japan), following the established method for quantifying SARA fractions. Colloidal instability indices (Equations (1)–(3)) as indicators of aging have been suggested by Feng et al. [10] and have been used by many researchers to highlight the chemical changes associated with the aging of asphalt binder.

(1) $$I_{p}=\frac{\mathrm{Resins}}{\mathrm{Asphaltenes}}$$

(2) $$I_{S}=\frac{\mathrm{Asphaltenes}}{\mathrm{Saturates}+\mathrm{Aromatics}+\mathrm{Resins}}$$

(3) $$I_{c}=\frac{\mathrm{Aromatics}+\mathrm{Resins}}{\mathrm{Asphaltenes}+\mathrm{Saturates}}$$

In addition, Fourier-transform infrared spectroscopy (FTIR) is a widely used technique for evaluating the chemical changes that occur in asphalt binders during aging of asphalt. By detecting functional groups such as carbonyl and sulfoxide, FTIR provides direct evidence of oxidative reactions that contribute to binder stiffening and reduced flexibility. Its ability to sensitively track these molecular transformations makes FTIR a valuable complement to rheological testing in understanding the mechanisms of asphalt degradation. FTIR spectroscopy has been considered an indication of the aging severity, as chemical changes due to binder oxidation can be tracked in some bands in the FTIR spectrum. Most studies have observed an increase in carbonyls (wavelength around 1700 cm^−1^) and sulfoxides (wavelength around 1030 cm^−1^), with some also indicating an increase in the aromatic band (around 1600 cm^−1^) [48,49,50]. A Thermo Nexus FTIR spectrophotometer (Nexus FT-IR, Thermo Fisher Scientific, Madison, WI, USA) equipped with Omnic software (version 6.2, Thermo Fisher Scientific, Madison, WI, USA) was used to acquire the FTIR spectra. Each specimen was scanned separately, and an average spectrum of each category was drawn to clarify the distinct effect of different aging procedures and disk positions on the binder. The wavenumber range scanned was from 4000 cm^−1^ to 400 cm^−1^. The aging effect was studied by following the changes of two characteristic infrared bands: the carbonyl band around 1700 cm^−1^ and the sulfoxide band around 1035 cm^−1^.

## 3. Results and Discussion

### 3.1. Dynamic Shear Rheometer Results

**Figure 4:**
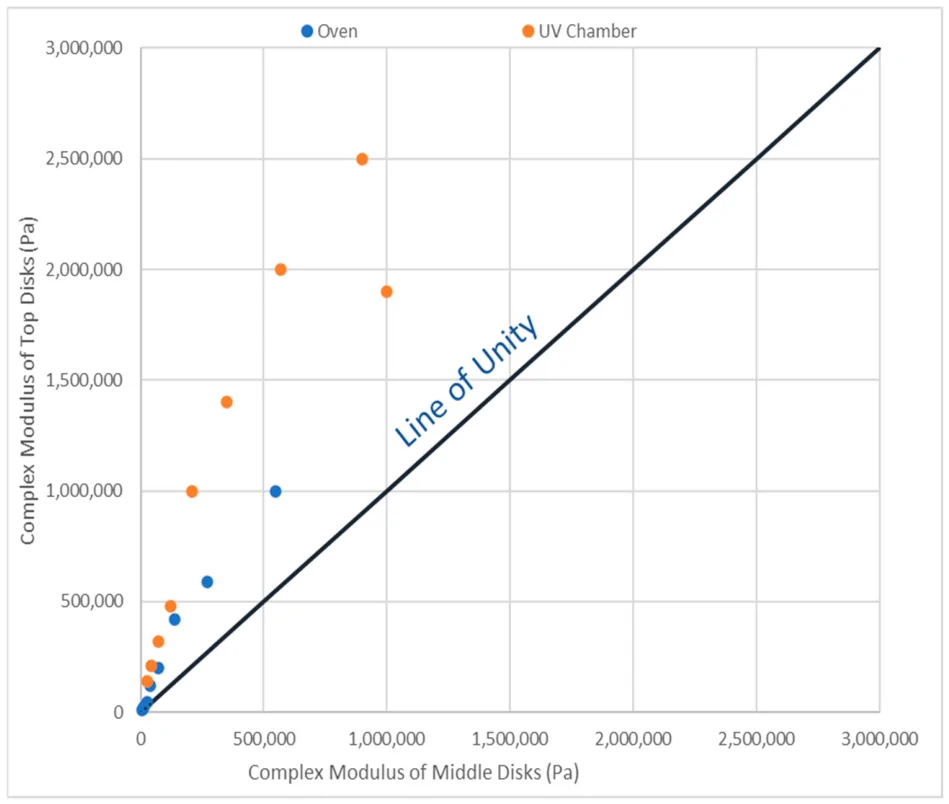
Temperature and average complex modulus at 1 Hz reference frequency.

Table 4 summarizes the ratio between top and middle disks for asphalt mixtures aged in an oven and UV chamber, and Table 5 summarizes the results and ratio between binder samples aged in an oven and a UV chamber. Finally, Table 6 compares the results from the top disks of aged asphalt mixture to the results from the aged binder specimens under the same aging conditions. Figure 4 presents the relationship between the complex moduli of extractions from middle disks and top disks of oven-aged and UV-aged mixtures at different temperatures. It can be noticed that all points are located above the equity line. Still, the results of the oven samples are closer to the equity line, compared to the results of the UV-aged samples, which indicates that the aging profile is steeper in UV-aged samples.

In terms of mixture aging, the results clearly show that the combined effect of UV light and heat is more significant than the effect of heat only. For example, the top disks were more severely aged than the middle disks, in both oven- and UV-aged samples. This can be explained in terms of the exposure of the surface disks to the environment, including heat and UV irradiation in the presence of oxygen. The ratio between UV top disk moduli and middle disk moduli ranged from 1.9 to 5.0, while the ratio in oven-aged samples ranged from 1.8 to 3.1, which also indicates that the top disks from the UV-aged samples were more aged compared to the middle disks and compared to the oven-aged samples. This supports the hypothesis that UV irradiation plays a catalytic role when absorbed by the pavement surface. In addition, the ratio between the UV-aged top disk and middle disk indicates that the UV irradiation can only penetrate a small thickness of the pavement, and most of the energy is absorbed by the top thin layer of the asphalt surface.

**Figure 5:**
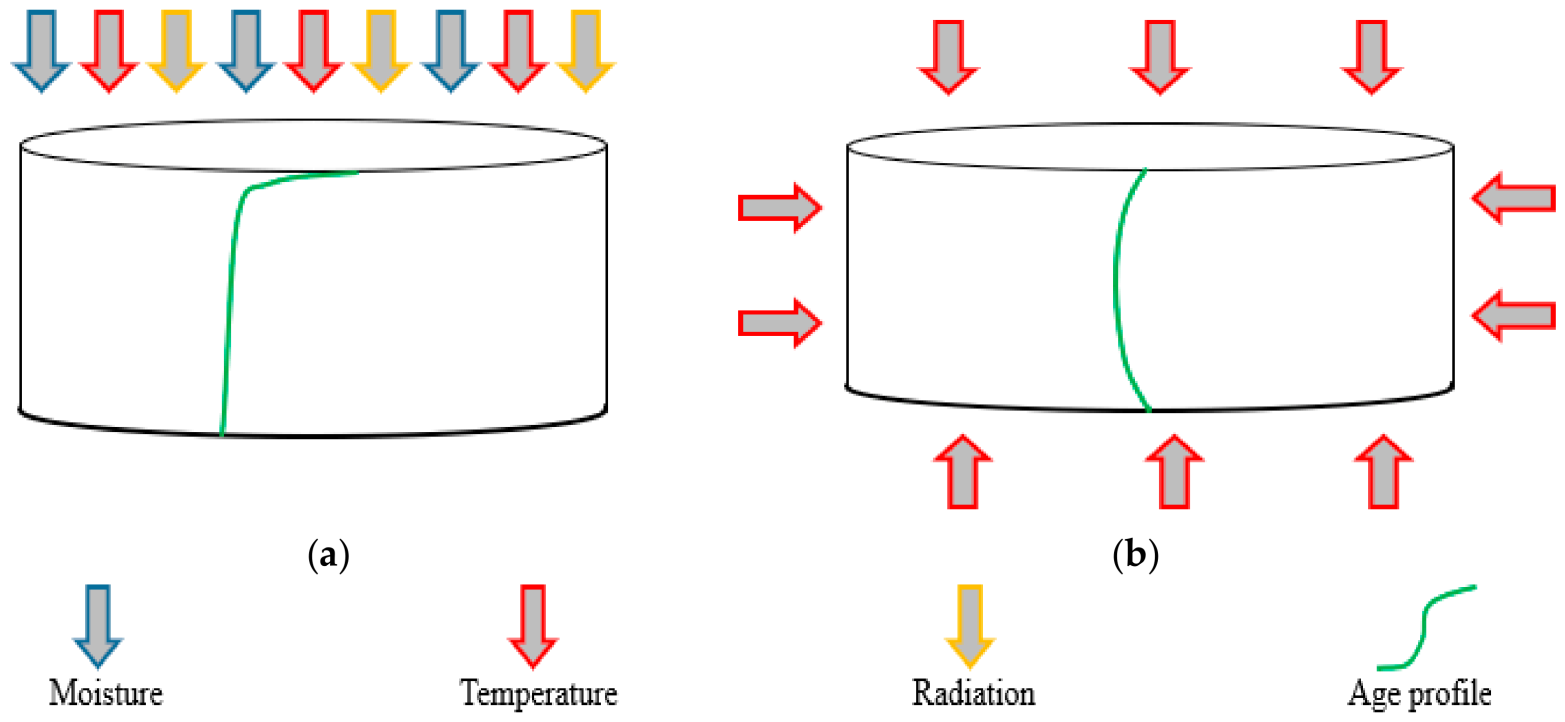
Schematic of asphalt aged in the (**a**) field and (**b**) a conventional oven [45].

White and Abouelsaad [46,51] hypothesized that the aging gradient in the case of oven conditioning is different from that of conditioning samples in a UV weathering chamber. This is because the heat affects cores from all directions, which are different from the actual field conditions in which samples are exposed to the environmental factors from the top, as shown in the Figure 5.

In terms of thin-film binder aging, UV aging caused more severe aging in terms of complex shear modulus, as the ratio between aged and unaged complex moduli ranged from 1.2 to 1.6 for the oven-aged samples. In contrast, the ratio ranged from 1.8 to 2.5 for the UV-aged thin film. Furthermore, the ratio between the UV-aged and oven-aged samples exceeded 1.0 at all temperatures, indicating that UV irradiation, in combination with heating of the thin films of binder, resulted in more severe aging. This is consistent with the mixture aging results.

A comparison between the effects of heat on asphalt mixtures and thin films of binder was also considered, with the ratio of the top disk to aged bitumen varying from 3.3 to 12.4 for oven-aged samples. In contrast, the same ratio for UV-aged samples ranged from 8.3 to 43.7. This indicates that the exposure of asphalt mixtures to UV irradiation for prolonged durations caused more severe aging than the aging of the thin-film binder specimens, under otherwise similar conditions. Similarly, for samples aged in an oven, a prolonged exposure of asphalt mixtures to heat caused more severe aging than the bituminous binder aged in an oven.

### 3.2. SARA Analysis Results

**Figure 6:**
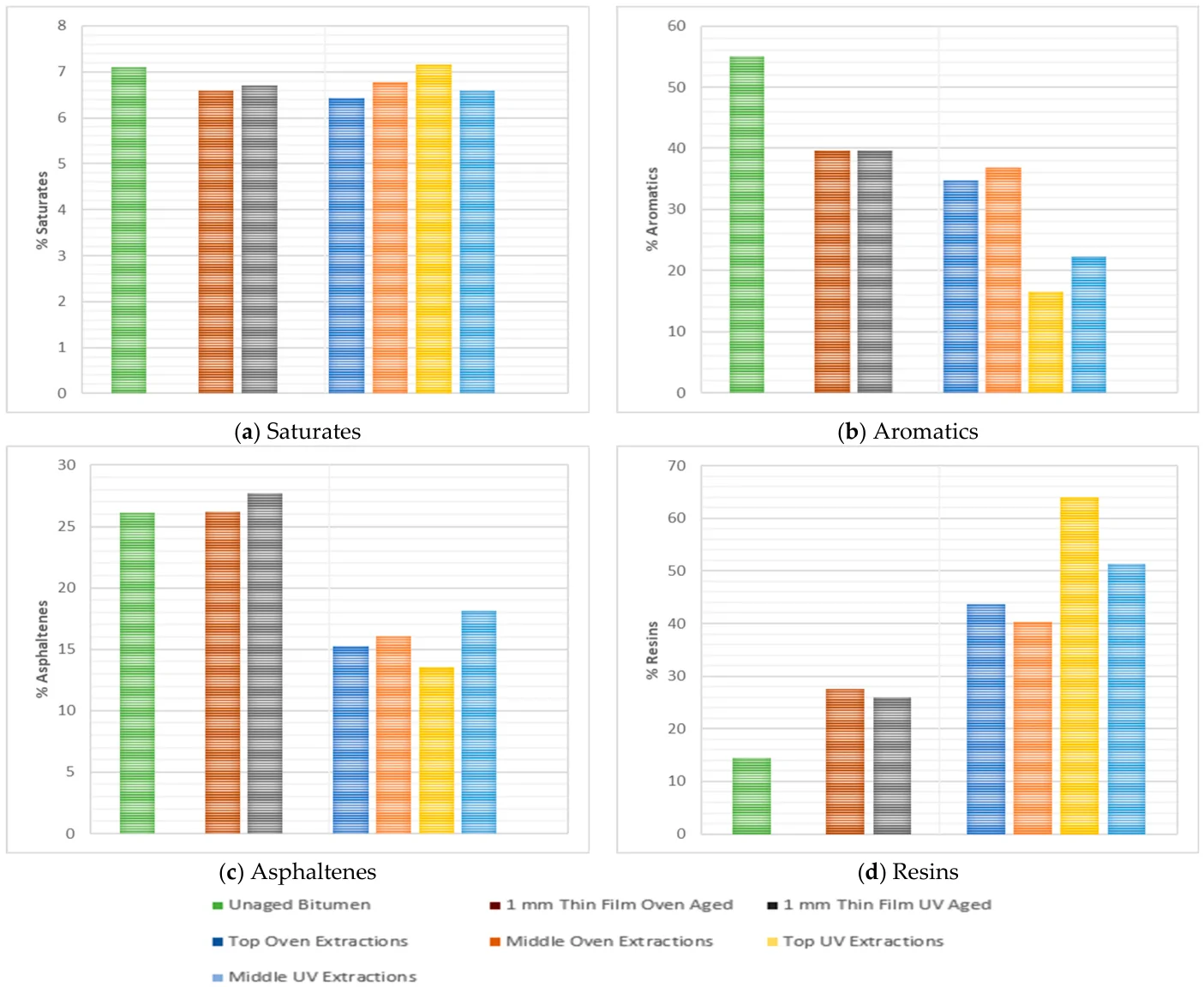
Average SARA fraction values of extractions from aged mixtures and SARA fractions of unaged bitumen and the oven- and UV-aged thin-film bituminous binder.

As previously explained, a SARA analysis of all extracted bitumen samples was performed. Table 7 and Table 8 summarize the results of the extractions from oven- and UV-aged samples, respectively, while Table 9 summarizes the results of oven- and UV-aged bituminous binder. The average results of the four fractions of extractions from asphalt mixtures and bituminous binder are shown in Figure 6.

Compared to the unaged binder, for asphalt mixtures, a significant reduction in aromatics and asphaltenes and a significant increase in resins were noticed, while saturates showed almost no difference. Table 10 summarizes the average difference between the different-aged samples and the unaged bitumen. For asphalt mixture top disks aged in the UV chamber, the highest difference from the unaged binder was observed, demonstrating the contribution of UV irradiation to the aging process of asphalt mixtures. In addition, the effect of pavement depth, in terms of the difference between thermal oxidation and photo-oxidation of asphalt mixtures, was more pronounced in the UV-aged compacted mixtures compared to the oven-aged asphalt mixtures. On the other hand, aged binder films showed almost no difference between thermal oxidation and photo-oxidation. Extractions from the top disks of asphalt mixture samples aged in the oven showed more severe aging compared to thin-film binder aged under the same conditions. Similarly, UV top disks showed more severe aging than the binder films aged under the same conditions.

**Figure 7:**
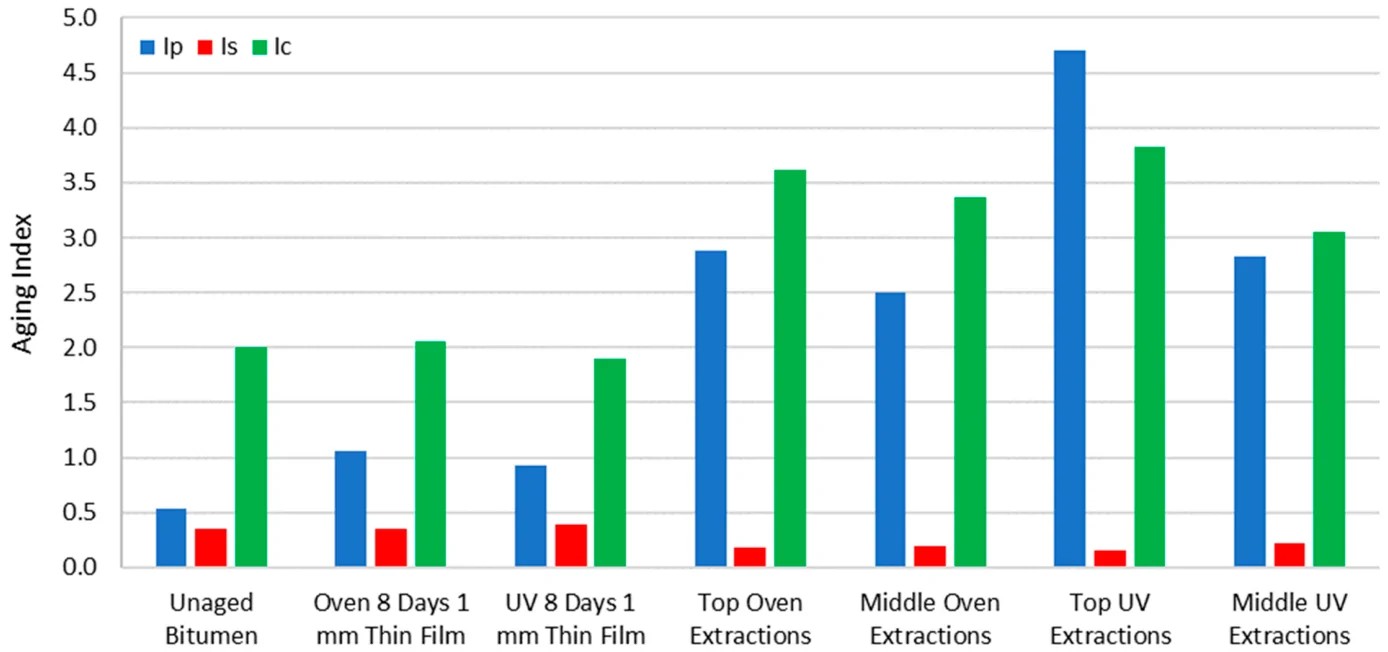
Colloidal indices of extractions from samples aged in the oven and UV chamber with reference to unaged bitumen and the oven-aged and UV-aged thin films.

A reduction in asphaltene content with aging was recorded for all extracted binder specimens. This is either because of the extraction process or the reactions between bitumen fractions and degraded polymers. As previously explained, researchers have suggested using colloidal instability indices to assess the aging behavior of binders. Figure 7 shows the changes in the colloidal instability indices of the unaged bitumen, oven-aged thin films, UV-aged thin films, and extractions from the top and the middle disks of both oven-aged and UV-aged samples.

It was concluded by Feng et al. [10] that aging reduces I_p_ and I_c_ while increasing I_s_, and these trends of increase and decrease were correlated with the increases in the penetration retention ratio (PRR), ductility retention ratio (DRR) and softening point. This was not the case in this study, because the highest I_p_ and I_c_ and the lowest I_s_ of extractions from the top disks of mixtures aged in the UV chamber showed the highest complex shear modulus. In contrast, the thin binder film aged in the UV chamber exhibited a higher complex modulus compared to the thin film aged in the oven but showed lower I_p_ and I_c_ values and higher I_s_ values. Hence, the trends of increasing or decreasing colloidal indices cannot be generalized and do not correlate to the rheological properties of bitumen. It is therefore better to evaluate the aging effects in terms of the absolute differences in the three reactive fractions, such as the asphaltenes, resins, and aromatics, as introduced in this study.

The general interpretation of the increasing and decreasing trends of colloidal indices previously suggested by Feng et al. [10] can be validated in neat liquid asphalt, which shows an increase in asphaltenes and resins fraction and loss in aromatics with aging. However, this is not the case in polymer-modified binder, as reported by Kleiziene et al. [43], who found a reduction in asphaltenes in polymer-modified binder, explaining this in terms of two probable scenarios. First, the polymer may be attributed to asphaltenes during the TLC-FID, or the SBS polymer may degrade due to aging, resulting in a reduction in the effective polymer content and, therefore, in the reported asphaltene fraction. Second, after aging, SBS polymers may combine with the asphaltenes into long chains with heavier molecular weight, making it harder to solute in toluene and pass through the chroma rod in the flame ionization detector used in the SARA analysis procedure. This would also under-report the asphaltene content. However, in this study, the loss in asphaltenes was recorded in the extracted binder only, while it was not recorded in the aged thin-film samples. This indicates two alternative explanation scenarios. First, we consider the different effects of heat and light, due to diffusion of binder between the aggregates, in contrast to the direct and aggressive effects of heat and light on the thin films. This may cause different cross-linked chemical reactions between the liquid asphalt molecules and the free radicals formed due to polymer degradation. From another point of view, the decline in asphaltene content may result from the binder extraction and recovery procedure, which includes leaving the loose mixture in toluene. This may result in a redistribution of the SARA fractions. Further research is required to understand the effects of binder recovery and the solvent on the measured SARA fractions and how this impacts neat liquid asphalt and polymer-modified binders differently.

### 3.3. FTIR Spectroscopy Results

**Figure 8:**
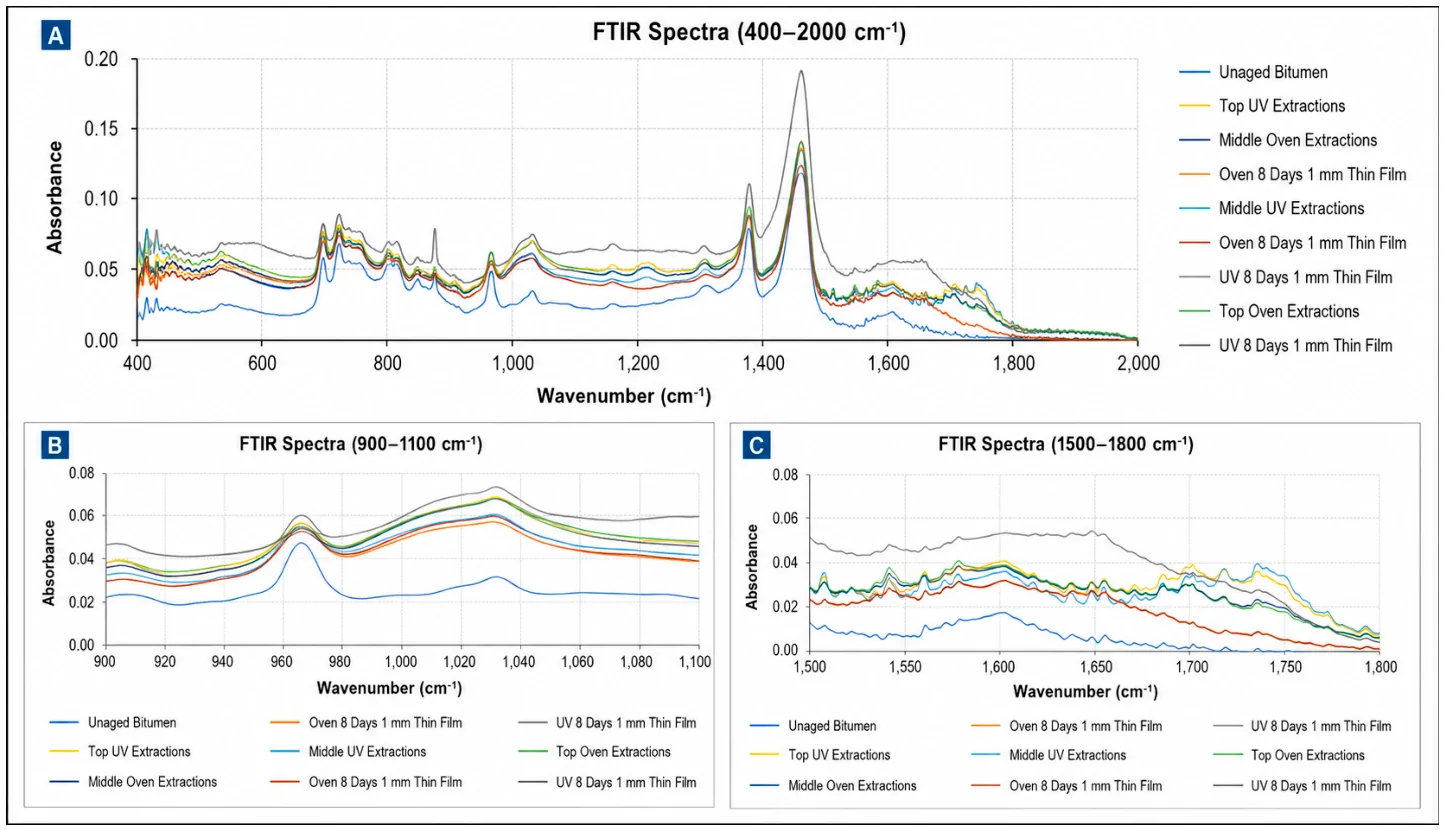
(**A**) Average FTIR spectra of extractions from samples aged in the oven and UV chamber with reference to unaged bitumen and the full spectra of oven-aged and UV-aged thin films. (**B**) Average FTIR spectra around sulphoxide peaks of extractions from samples aged in the oven and UV chamber with reference to unaged bitumen and the full spectra of oven-aged and UV-aged thin films. (**C**) Average FTIR spectra around carbonyl peaks and the main aromatic band of extractions from samples aged in the oven and UV chamber, with reference to unaged bitumen and the full spectra of oven-aged and UV-aged thin films.

**Figure 9:**
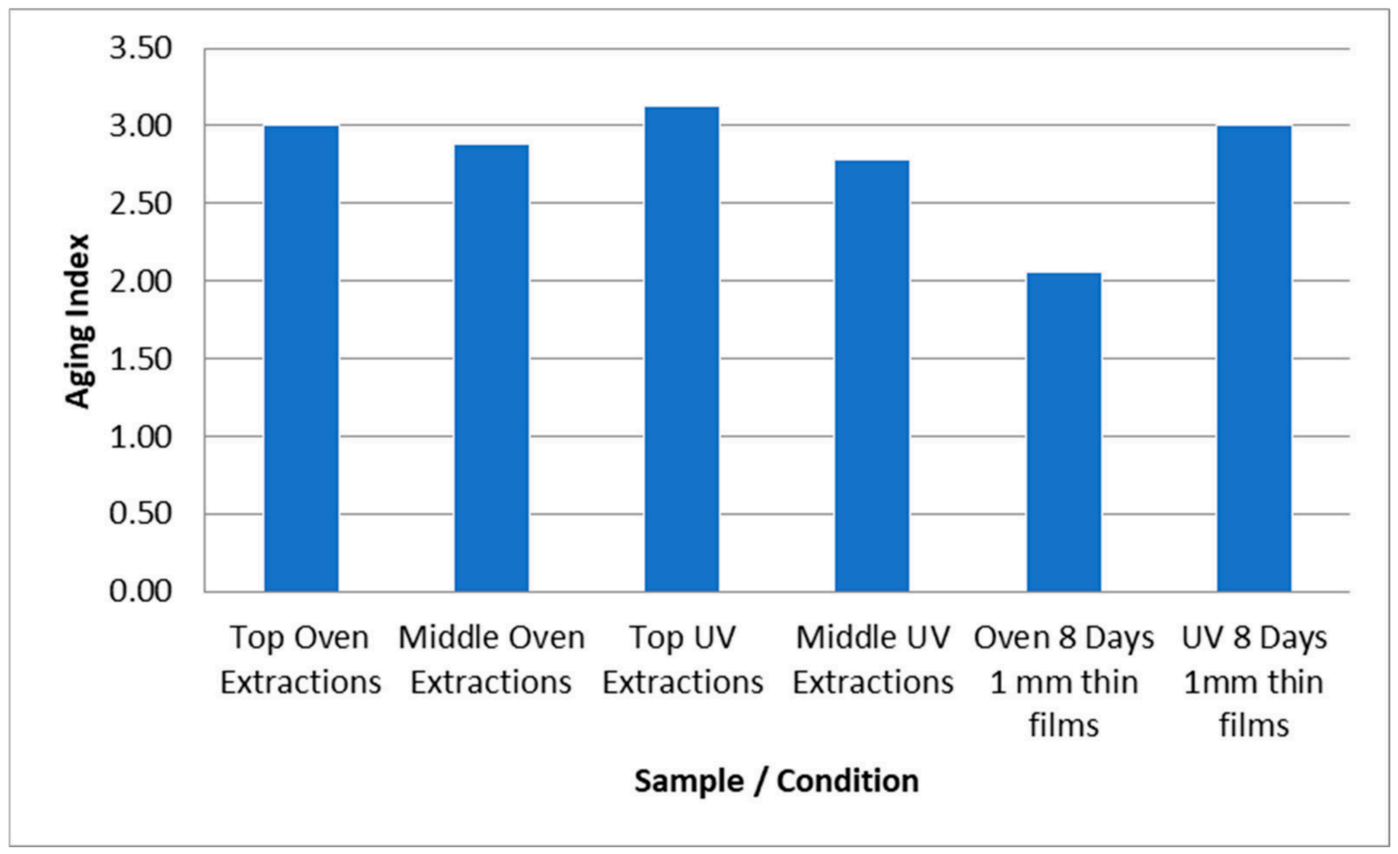
Aging index from FTIR analysis of extractions from samples aged in the oven and UV chamber with reference to unaged bitumen and the oven-aged and UV-aged thin films.

Extracted bitumen, aged bitumen, and unaged bitumen samples were analyzed using FTIR, and the average spectrum of extractions from the aged mixture disks was calculated as an average of absorption at every wavelength. Figure 8A shows the average spectra of extractions from aged mixtures with reference to the unaged binder, in addition to the oven-aged and UV-aged thin films. Figure 8B and Figure 8C show the spectra around the sulfoxide band and around the carbonyl and aromatic bands together, respectively. It is clear from the graphical representations that all samples of extracted binder from aged mixtures, as well as aged binder films, showed an increase in the absorbance of sulphoxides, carbonyls, and aromatics. Table 11 summarizes the carbonyl and sulphoxide peak values. The summation of carbonyl and sulphoxide peak values can be considered as an indicator of the severity of aging, as suggested in multiple previous studies. An aging index can then be calculated as the ratio between the summation of carbonyls and sulphoxides of an aged sample and the summation of carbonyls and sulphoxides of unaged binder. Figure 9 shows the values of the aging indices of different samples, which can be used to compare the severity of aging between different-aged samples.

From the aging index values, it can be noticed that the top UV-aged disks showed the highest degree of aging, compared to all other samples. In terms of comparing thermal oxidation and photo-oxidation, the aging indices of UV-aged samples were all higher than the corresponding values of the samples that were aged in the oven, which again demonstrates the contribution of UV irradiation to the aging process. In terms of the asphalt mixtures, FTIR results followed the same trend of previously discussed indicators, as the top disks were more aged than the middle disks, with ratios of 1.04 and 1.12 for oven-aged samples and UV-aged samples, respectively. This also demonstrates the effect of UV irradiation being limited to the thin uppermost portion of a pavement surface layer. In addition, thermal-oxidative aging of the bituminous binder for 8 days showed less severe aging than the extracted bitumen from the top and middle disks of the samples that were aged in the oven. Similarly, the UV-aged bituminous binder showed less severe aging than the extractions from the top disks of the UV-aged asphalt mixtures. From another perspective, the ratio between the aging index of top disks and the corresponding aged thin-film binder was 1.46 and 1.04 in the case of thermal-oxidative aging and photo-oxidative aging, respectively. This indicates that UV aging of both bituminous binder and asphalt mixtures accelerates the formation of the carbonyls and the sulphoxides. Again, this is consistent with the DSR analysis findings.

On the other hand, the previously reported differences in SARA analysis results between extractions from aged asphalt mixtures and aged thin binder film specimens can be compared to the FTIR analysis results. The spectra of recovered binder from asphalt mixtures showed some shifts from the spectra of aged thin films of binder, as the formation of carbonyls dominated in the spectra of recovered bitumen. At the same time, the main aromatic band peaks were clearer in the aged bituminous binder films. This may mean that different chemical reactions occur in the case of asphalt mixture aging compared to bitumen aging. Further investigations are recommended to better understand the nature of chemical reactions that take place during aging in both neat and modified asphalt binder.

### 3.4. Comparative Analysis of SARA, DSR, and FTIR Findings

The results from the SARA analysis correlate with the DSR findings in several key areas, although there are differences in the aging processes of the materials. Both analyses show that UV aging has a more substantial impact on asphalt mixtures than oven aging. SARA results indicate a notable decrease in aromatics and asphaltenes, alongside an increase in resins, for UV-aged asphalt mixtures, suggesting significant chemical changes due to UV exposure. This matches the DSR results, which indicate that UV aging leads to a more pronounced increase in complex shear modulus, indicating greater stiffness. Additionally, the SARA analysis highlights a larger contrast between the top and middle layers of UV-aged samples, emphasizing the significant impact of UV irradiation on surface layers of asphalt concrete, which the DSR confirmed by showing that UV-aged samples exhibit higher modulus ratios than those aged in the oven. FTIR analysis adds further evidence by showing higher aging indices for UV-aged samples, suggesting more oxidation and greater formation of carbonyls and sulphoxides. These findings are consistent with SARA and DSR analyses, demonstrating that UV aging accelerates these chemical transformations. However, the FTIR results also reveal a shift in chemical composition, with carbonyl formation being more noticeable in the spectra of recovered bitumen from asphalt mixtures than from thin films. This suggests that different aging processes occur in asphalt mixtures compared to binder films, which warrants further study. Nevertheless, the FTIR analysis of aged thin films showed comparable results with extracted binders, reinforcing the reliability of these findings.

## 4. Conclusions

The results of this study demonstrate that UV irradiation plays a significant role in accelerating the aging process of compacted asphalt mixtures under controlled laboratory conditions, as evidenced by changes in the complex modulus, SARA fractions, and FTIR functional indices. Under the tested laboratory exposure, the depth-wise aging profile was more pronounced in asphalt mixture specimens subjected to combined UV radiation and heat compared to those subjected to heat only. This indicates that incorporating UV irradiation alongside heat captures surface-level photo-oxidative mechanisms that traditional thermal-only conditioning omits.

Hence, based on the specific experimental conditions, materials, and specimen geometries investigated, the following conclusions and practical recommendations are drawn:

- Targeted Specimen Sectioning: For laboratory evaluations focused on surface-bound distresses (such as surface erosion, fretting, and raveling), cutting compacted asphalt mixtures into thin disks and aging the top disk provides a practical testing approach that mitigates the physical specimen distortion observed in full-thickness core samples.
- Accounting for Aggregate Energy Diffusion: Evaluating binders extracted from aged mixture cores accounts for energy diffusion between the aggregate matrix and the bitumen phase. Under the conditions evaluated, testing mixture-extracted binders avoids the aggressive, unshielded exposure of thin binder films, which can induce severe localized reactions—particularly in polymer-modified binders experiencing concurrent binder oxidation and polymer degradation.
- Accelerated Protocol Development: Including UV irradiation in laboratory aging protocols is recommended when evaluating surface-bound degradation pathways in bituminous binders and asphalt mixtures.
- Future Work: Further research is recommended to investigate the specific differences in chemical degradation mechanisms occurring in neat asphalt binders versus polymer-modified binders during combined UV and thermal exposure.

## Figures and Tables

**Table 1:** Volumetric properties of DGA mixtures [42].

Property	Units	Test Method	Value
VMA	% (by volume)	AS/NZS 2891.8	14.4
VFB	% (by volume)	AS/NZS 2891.8	76
Air Voids	% (by volume)	AS/NZS 2891.8	4.2
Binder Content	% (by mass)	AS/NZS 2891.8	5.4
Binder Type	N/A	Selected by the designer	A10E
Stability	kN	AS/NZS 2891.5	13.2
Flow	mm	AG: PT/T231	3.7
Tensile Strength	MPa	AG: PT/T232	1036
Resilient Modulus	MPa	AS/NZ 2891.13.1	1870
TSR	%	AG: PT/T232	87

**Table 2:** Bituminous binder properties [42].

Property	Units	Test Method	Value
Softening Point	°C	AG: PT/T131	96
Torsional Recovery at 25 °C	%	AG: PT/T131	66
Viscosity at 165 °C	Pa.s	AG: PT/T131	0.693
Performance Grade	°C (at traffic level)	AASHTO TP 70	82 (Extreme)
Elastic Recovery	% (82 °C, 3.2 kPa)	AASHTO TP 70	96

**Table 3:** Weathering chamber conditions [45].

Characteristic	Setting
Irradiance	50 W/m^2^
Irradiance Control	300–400 nm
Chamber Temperature	70 °C
Humidity %	0%
Moisture Spray	Off

**Table 4:** Average complex modulus values and ratios between extractions from top and middle disks aged in oven and UV chamber.

Temperature	Top Oven Extractions	Middle Oven Extractions	Top/Middle Ratio Oven	Top UV Extractions	Middle UV Extractions	Top/Middle Ratio UV
30	1.0 × 10^6^	5.5 × 10^5^	1.8	1.9 × 10^6^	1.0 × 10^6^	1.9
35	5.9 × 10^5^	2.7 × 10^5^	2.1	2.5 × 10^6^	9.0 × 10^5^	2.8
40	4.2 × 10^5^	1.4 × 10^5^	3.1	2.0 × 10^6^	5.7 × 10^5^	3.5
45	2.0 × 10^5^	7.1 × 10^4^	2.8	1.4 × 10^6^	3.5 × 10^5^	4.2
50	1.2 × 10^5^	3.8 × 10^4^	3.0	1.0 × 10^6^	2.1 × 10^5^	5.0
55	4.6 × 10^4^	2.6 × 10^4^	1.8	4.8 × 10^5^	1.2 × 10^5^	4.0
60	2.8 × 10^4^	1.5 × 10^4^	1.8	3.2 × 10^5^	7.2 × 10^4^	4.4
65	1.7 × 10^4^	9.2 × 10^3^	1.9	2.1 × 10^5^	4.4 × 10^4^	4.8
70	1.1 × 10^4^	5.6 × 10^3^	2.0	1.4 × 10^5^	2.7 × 10^4^	5.2

**Table 5:** Average complex moduli and ratios between oven-aged, UV-aged, and unaged thin bitumen films [45].

Temperature	Unaged Binder	Oven-Aged Thin Film	UV-Aged Thin Film	Oven/Unaged Ratio	UV/Unaged Ratio	UV/Oven Ratio
30	1.1 × 10^5^	1.2 × 10^5^	2.3 × 10^5^	1.2	2.1	1.8
35	5.4 × 10^4^	6.3 × 10^4^	1.2 × 10^5^	1.2	2.3	1.9
40	2.8 × 10^4^	3.4 × 10^4^	6.8 × 10^4^	1.2	2.4	2.0
45	1.6 × 10^4^	2.0 × 10^4^	3.9 × 10^4^	1.3	2.5	1.9
50	1.3 × 10^4^	1.6 × 10^4^	2.4 × 10^4^	1.3	1.8	1.5
55	6.4 × 10^3^	1.1 × 10^4^	1.6 × 10^4^	1.6	2.5	1.5
60	4.5 × 10^3^	7.1 × 10^3^	1.1 × 10^4^	1.6	2.4	1.5
65	3.2 × 10^3^	4.9 × 10^3^	7.3 × 10^3^	1.5	2.3	1.5
70	2.3 × 10^3^	3.5 × 10^3^	5.1 × 10^3^	1.5	2.2	1.5

**Table 6:** Average complex modulus values and ratios between extractions from top disks and thin films of aged bituminous binder under the same conditions.

Temperature	Top Oven Extractions	Oven-Aged Thin Film	Top Disc/Thin Film Oven Ratio	Top UV Extractions	UV-Aged Bitumen Thin Film	Top Disc/Thin Film UV Ratio
30	1.0 × 10^6^	1.2 × 10^5^	8.1	1.9 × 10^6^	2.3 × 10^5^	8.3
35	5.9 × 10^5^	6.3 × 10^4^	9.3	2.5 × 10^6^	1.2 × 10^5^	20.3
40	3.4 × 10^5^	3.4 × 10^4^	12.4	2.0 × 10^6^	6.8 × 10^4^	29.1
45	2.0 × 10^5^	2.0 × 10^4^	9.8	1.4 × 10^6^	3.9 × 10^4^	36.8
50	1.2 × 10^5^	1.6 × 10^4^	7.2	1.0 × 10^6^	2.4 × 10^4^	43.7
55	4.8 × 10^4^	1.1 × 10^4^	4.3	4.8 × 10^5^	1.6 × 10^4^	30.1
60	2.9 × 10^4^	7.1 × 10^3^	3.9	3.2 × 10^5^	1.1 × 10^4^	29.3
65	1.8 × 10^4^	4.9 × 10^3^	3.6	2.1 × 10^5^	7.3 × 10^3^	28.3
70	1.2 × 10^4^	3.5 × 10^3^	3.3	1.4 × 10^5^	5.1 × 10^3^	27.7

**Table 7:** SARA analysis of oven-aged mixture extractions [45].

Replicate/Disk	% Saturates	% Aromatics	% Resins	% Asphaltenes
1/Top	6.2	44.2	36.1	13.5
1/Middle	6.3	44.2	32.5	17
2/Top	6.7	28.7	50.2	14.4
2/Middle	6.8	31.6	46.7	14.9
3/Top	6.4	31.1	44.7	17.8
3/Middle	7.2	34.7	41.8	16.3

**Table 8:** SARA analysis of UV-aged mixture extractions [45].

Replicate/Disk	% Saturates	% Aromatics	% Resins	% Asphaltenes
1/Top	7.0	15.6	62.6	14.8
1/Middle	6.6	21.8	52.6	19
2/Top	7.1	16.1	63.1	13.7
2/Middle	6.5	22.3	53.1	18.1
3/Top	7.2	16.3	62.9	13.6
3/Middle	6.7	22.5	52.3	18.5

**Table 9:** SARA analysis results of unaged bitumen and oven- and UV-aged bituminous binder [45].

Replicate/Disk	% Saturates	% Aromatics	% Resins	% Asphaltenes
Unaged bitumen	7.1	53	13.8	26.1
Oven 1 mm thin film	6.6	39.7	27.6	26.2
UV 1 mm thin film	6.7	39.7	25.9	27.8

**Table 10:** Difference in aromatics, resins, and asphaltenes from unaged bitumen.

Sample	Difference in SARA Fractions		
	% Aromatics	% Resins	% Asphaltenes
Top Oven Extractions	−18.3%	+29.9%	−10.9%
Middle Oven Extractions	−16.2%	+26.5%	−10.0%
Top UV Extractions	−37.6%	+50.1%	−12.5%
Middle UV Extractions	−29%	+37.5%	−8.0%
Oven 8 Days Thin Film	−13.3%	+13.8%	+0.1%

**Table 11:** Carbonyl and sulphoxide peaks of extractions from samples aged in the oven and UV chamber with reference to unaged bitumen and the oven-aged and UV-aged thin films.

Sample	Carbonyls	Sulphoxides	Carbonyls + Sulphoxides
Unaged Bitumen	0.004	0.029	0.033
Top Oven Extractions	0.030	0.069	0.099
Middle Oven Extractions	0.030	0.065	0.095
Top UV Extractions	0.037	0.066	0.103
Middle UV Extractions	0.033	0.059	0.092
Oven 8 Days thin films	0.013	0.055	0.068
UV 8 Days thin films	0.034	0.065	0.099

## Data Availability

The original contributions presented in this study are included in the article. Further inquiries can be directed to the corresponding author.
